# Day patient treatment for traumatic grief: preliminary evaluation of a one-year treatment programme for patients with multiple and traumatic losses

**DOI:** 10.1080/20008198.2017.1375335

**Published:** 2017-09-04

**Authors:** Annemiek de Heus, Sophie M. C. Hengst, Simone M. de la Rie, A. A. A. Manik J. Djelantik, Paul A. Boelen, Geert E. Smid

**Affiliations:** ^a^ Foundation Centrum ’45, Diemen, The Netherlands; ^b^ Arq Psychotrauma Expert Group, Diemen, The Netherlands; ^c^ Department of Clinical Psychology, Utrecht University, Utrecht, The Netherlands

**Keywords:** Traumatic loss, bereavement, PTSD, depression, day patient treatment, brief eclectic psychotherapy, refugees, multidisciplinary, ongoing stressors

## Abstract

**Background**: Bereaved individuals who have lost a loved one under traumatic circumstances can develop symptoms of Persistent Complex Bereavement Disorder (PCBD) and/or Posttraumatic Stress Disorder (PTSD). This is particularly common in refugees, as they frequently have been confronted with multiple traumatic losses. For patients with severe PTSD and traumatic grief a treatment programme was developed, embedding individual traumatic grief focused therapy in a group-based multidisciplinary day patient treatment programme. The day patient treatment comprised a weekly five-hour programme consisting of three phases with a duration of four months each.

**Objective**: To evaluate the feasibility and potential effectiveness of the treatment programme.

**Method**: Data were analyzed from 16 participants treated between October 2013 and March 2014. PTSD severity and PTSD/PCBD diagnoses were measured during the initial and final phases of treatment using the Clinician-Administered PTSD Scale for DSM-IV (CAPS) and the Traumatic Grief Inventory Self Report (TGI-SR). One clinical case is presented in more detail. Treatment attendance was also registered and therapist satisfaction was evaluated in a focus group.

**Results**: Thirteen patients (81%) completed the treatment. Each day of the treatment programme was attended by a mean of 76% of the participants. In the focus group, therapists noted symptom reduction in their patients and they therefore regarded Brief Eclectic Psychotherapy for Traumatic Grief (BEP-TG) as an effective therapy for their patients. During treatment, significant decreases in PTSD severity as well as diagnosable PTSD and PCBD were observed.

**Conclusions**: Results support the feasibility and potential effectiveness of the day patient treatment programme for traumatic grief. The programme appears to be particularly suitable for refugees with severe PTSD and PCBD psychopathology, who may not benefit enough from usual care.

## Abstract (es)

**Planteamiento:** Las personas en duelo que han perdido a un ser querido en circunstancias traumáticas pueden desarrollar síntomas del trastorno por duelo complejo persistente (TDCP) y/o trastorno por estrés postraumático (TEPT). Esto es particularmente habitual en refugiados, ya que con frecuencia se han enfrentado a múltiples pérdidas traumáticas. Se desarrolló un programa de tratamiento para pacientes con TEPT grave y duelo traumático que incluía terapia individual centrada en el duelo traumático dentro de un programa grupal multidisciplinario de tratamiento de día. El tratamiento de día consistía en un programa semanal de cinco horas que constaba de tres fases, con una duración de cuatro meses cada una.

**Objetivo:** Evaluar la viabilidad y la eficacia potencial del programa de tratamiento.

**Métodos:** Se analizaron los datos de dieciséis participantes tratados entre octubre de 2013 y marzo de 2014. Se midió la gravedad del TEPT y los diagnósticos de TEPT / TDCP durante las fases inicial y final del tratamiento utilizando la Escala de TEPT administrada por el clínico para el DSM-IV (CAPS, siglas en inglés de *Clinician-Administered PTSD Scale for DSM-IV*) y el Autoinforme del inventario de duelo traumático (TGI-SR, siglas en inglés de *Traumatic Grief Inventory Self Report*). Se presenta con más detalle un caso clínico. También se registró la asistencia al tratamiento y se evaluó la satisfacción del terapeuta en un grupo focal.

**Resultados:** Trece pacientes (81%) completaron el tratamiento. A cada día del programa de tratamiento asistió una media del 76% de los participantes. En el grupo focal, los terapeutas observaron reducción de síntomas en sus pacientes y, por lo tanto, consideraron la psicoterapia ecléctica breve para el duelo traumático (BEP-TG, siglas en inglés de *Brief Eclectic Psychotherapy for Traumatic Grief*) como una terapia eficaz para sus pacientes. Durante el tratamiento, se observaron reducciones significativas de la gravedad del TEPT, así como de los diagnósticos de TEPT y TDCP.

**Conclusión:** Los resultados apoyan la viabilidad y la eficacia potencial del programa de tratamiento de día para duelo traumático. El programa parece ser particularmente adecuado para refugiados con una psicopatología grave de TEPT y TDCP, que pueden que no se beneficien lo suficiente de asistencia médica frecuente.

## Abstract (zh)

**背景：**在创伤情景下失去所爱之人后，丧亲者会出现长期复杂丧亲障碍（PCBD）以及/或者创伤后应激障碍（PTSD）。这在难民中尤其常见，因为难民经常要面对多次创伤性丧失。对有严重PTSD和创伤性伤痛的病人我们开发了一个治疗方案，在针对多科日间留院病人的团体治疗方案中整合了以个人创伤性伤痛为中心的疗法。这个日间留院病人治疗方案包括每周5小时的治疗，一共三个阶段，持续四个月。

**目标：**评估治疗方案的可行性和潜在效果。

**方法：**使用16个参加者在2013年10月和2014年3月之间的治疗数据进行分析。治疗开始和结束阶段的PTSD严重程度和PTSD/PCBD诊断使用临床用DSM-IV PTSD量表（CAPS）和创伤性伤痛自评清单（TGI-SR）。并对其中一个临床案例的更多细节进行报告。治疗参与度和治疗师满意度在一个焦点小组（focus group）里进行评估。

**结果：**13个病人（81%）完成了治疗。平均76%的病人参加了每天的治疗过程。在焦点小组中，治疗师记录到病人的症状减轻，因此创伤性伤痛的简明折衷心理疗法（BEP-TG）可作为一种有效的治疗。在治疗过程中，PTSD的严重程度显著降低，PTSD和PCBD的诊断率也显著减少。

**结论：**结果支持了这个针对创伤性伤痛的日间留院病人治疗方案的可行性和潜在效果。这个方案表现出对有严重PTSD和PCBD的难民尤其合适，而他们在普通的治疗中可能效果甚微。

Traumatic experiences often include the loss of a loved one. Traumatic experiences in which a loved one has died may lead to severe psychiatric problems in bereaved individuals, such as Posttraumatic Stress Disorder (PTSD), Persistent Complex Bereavement Disorder (PCBD) and Major Depression Disorder (MDD) (Boelen, Reijntjes, Djelantik, & Smid, 2016; Nickerson et al., 2014). Treatments for this group of bereaved individuals are mostly focused on PTSD and hardly address grief specifically (Smid et al., 2015).

*Traumatic loss* refers to the loss of one or several close family members or friends due to homicide, suicide or accident (Currier, Holland, & Neimeyer, 2006). *Traumatic grief (TG)* will be defined in this study as a diagnosis of persistent complex bereavement disorder (PCBD) and/or (symptoms of) posttraumatic stress disorder (PTSD) following a traumatic loss (Barle, Wortman, & Latack, 2015). Core symptoms of PCBD include yearning and longing for the deceased, intense emotional pain and preoccupation with the deceased or the circumstances of the death (American Psychiatric Association [A.P.A.], 2013).

Refugees are especially vulnerable to traumatic grief, given that they are often exposed to a variety of traumatic events and traumatic loss. They are often faced with multiple traumatic losses and/or ambiguous loss(es), i.e. the loss of a loved one who is missing and often presumed dead but whose remains have never been found (Boss, 2002). Studies have shown that traumatic losses in this group can lead to both PCBD and PTSD (Momartin, Silove, Manicavasagar, & Steel, 2004; Nickerson et al., 2014; Stammel et al., 2013). Comorbidity of PCBD and/or PTSD with other psychiatric disorders such as major depressive disorder (MDD) (Kessler, Sonnega, Bromet, Hughes, & Nelson, 1995; Schaal, Dusingizemungu, Jacob, Neuner, & Elbert, 2012), psychotic disorders (Sareen, Cox, Goodwin, & Asmundson, 2005) and substance abuse disorders (Kessler et al., 1995) is often observed. Furthermore, refugees are confronted with ongoing stressors that can fuel the risk for psychopathology (Laban 2004, 2005), such as insecurity about residential status, concerns about wellbeing of family members left behind, socioeconomic disadvantage, and adaptation to the host culture.

So far, treatment of mental health problems of refugees has mainly focused on PTSD and not on PCBD (Nickerson, Bryant, Silove, & Steel, 2011). Only few controlled studies regarding trauma-focused treatments in refugees have been done (Nickerson et al., 2011). A recent study within our research group examining treatment response among multiple traumatized patients indicated that asylum seekers/temporary refugees and resettled refugees showed lower PTSD symptom reduction compared with patients with profession-related PTSD, specifically police officers and veterans. Moreover, severity of symptoms dropped but still remained high (Ter Heide & Smid, 2015).

The day patient treatment programme described in this article was developed in a Dutch mental health care institute specialized in the treatment of traumatized populations. The institute offers individual treatment for outpatients as well as day patient and inpatient treatment. For patients with complex mental health problems, individual outpatient care is often not sufficient since it does not cover all areas in which support and treatment is needed. We developed a multidisciplinary group-based day patient treatment programme for refugees with complex mental health problems, suffering from PTSD, PCBD and/or MDD, ongoing stressors, such as mentioned above, and with limited daily activities and social contacts, who did not improve enough from outpatient care. First, with the development of this treatment we aimed to address the complexity and the severity of the symptoms of traumatic grief by offering specialized treatment for a population of patients who suffered multiple traumatic losses and traumatic experiences, and may not benefit enough from regular PTSD treatment. Second, we wanted to offer support in strengthening social networks and social activation, work, education and legal issues. Because of the severity of symptoms within this patient group, and multiple treatment goals in different problem areas, the day patient treatment comprised a one-year weekly five-hour programme. Based on the guidelines for complex PTSD (Cloitre et al., 2012) and guideline recommendations for PTSD in refugees (National Institute for Clinical Excellence, 2005), the treatment consisted of three phases. Each phase started with a group of eight patients and had a duration of four months.

The focus of *phase 1* is on stabilizing the psychiatric symptoms and investing in mutual trust and support within the group. This is obtained by offering group therapy, consisting of psycho-education about traumatic grief and PTSD along with explanation and practising of stabilizing techniques. Also, much attention goes to the consequences of traumatic grief and PTSD for interpersonal/family relationships, self-esteem and functioning in society and work. A group counsellor creates and maintains a therapeutic climate, focuses on the current state of the patients, provides emotional support, and offers suggestions on how to improve daily functioning. Through art therapy patients are encouraged to share experiences (both positive and negative) and to work with creative symbols as a way of expressing emotions. Through psychomotor therapy, patients are helped to gain control over emotional instability and somatic signals of distress. Individual therapist and physician consultations are offered regularly, aimed at stabilization and treating comorbid psychiatric disorders, e.g. MDD and/or mild substance abuse, and, if indicated, medication is prescribed conforming to the guidelines for PTSD and MDD.

In *phase 2*, the focus is on emotionally and cognitively processing the loss (mourning and memory-processing), through individual Brief Eclectic Psychotherapy for Traumatic Grief (BEP-TG) (Smid et al., 2015). The 16-session BEP-TG protocol, of which each session within the day patient treatment context comprises 75 minutes, consists of three stages. The first stage, information and motivation, provides an introduction to the treatment for the patient and close others. Characteristics of the traumatic loss are discussed and basic information is provided on cognitive processing and attachment reactions. The second stage includes grief-focused exposure, writing assignments and mementos and aims at integrating the memory of the traumatic loss, and finding alternatives to attempting to avoid distress. The third stage, finding meaning, activation and farewell ritual, aims to resolve maladaptive negative appraisals of the traumatic loss and further diminishing sensitivity to matching triggers and new stressors. During phase 2, group therapy and art therapy are maintained to enhance support and facilitate emotional processing. *Phase 3* focuses on resocialization, by means of group therapy sessions in which concrete individual goals are addressed, together with a social worker and a group counsellor. Patients are encouraged to discuss future plans and strengthen social networks. They are assisted in applying for jobs or voluntary work and expanding their fields of interest.

In this article, experiences with the first 16 consecutive patients referred to the programme are presented. The purpose of this study was twofold; first, to evaluate the feasibility of the day patient treatment for this particular population and, second, to describe the potential effectiveness in terms of the change in PTSD severity as well as diagnoses of PCBD and PTSD during the treatment.

## 1. Methods

### 1.1. Setting

As a part of ongoing innovation of specialized care, the day patient treatment was implemented at Foundation Centrum ’45, a Dutch mental health institute specialized in the treatment of populations traumatized by war, organized violence and work-related violence. Centrum ’45 receives national referrals of patients who, due to their complex psychopathology, cannot be treated in general mental healthcare, or who have shown insufficient response to treatment in general mental healthcare.

### 1.2. Participants and procedure

Participants were 16 consecutive patients, referred to the group based day patient treatment for traumatic grief between October 2013 and February 2014, after being clinically diagnosed with PTSD and PCBD. Participants who enrolled in October 2013 started the individual BEP-TG treatment in January 2014 and the second group started in June 2014. The BEP-TG had a duration of 16 sessions and was offered by trained therapists (i.e. psychologists and psychiatrists). This group of 10 therapists who delivered the BEP-TG were all experienced in rendering different kinds of treatments to refugees with complex mental health problems. Regular supervision about the therapy process was provided by a professor and clinical psychologist specialized in the treatment of traumatic grief (PB). Patients with insufficient Dutch language proficiency, acute and active suicidality, severe psychotic symptoms and/or severe alcohol or substance abuse were not eligible for the treatment. Psychiatric comorbidity was assessed by a psychiatrist or a licensed clinical psychologist. Standardized measurements were performed within the first two months of phase 1 of the day patient treatment programme and within the last two months of phase 3 by a team of independent trained psychologists and psychiatrists. All followed a diagnostic training and regular supervision of the diagnostic measurements was provided by senior psychologists. All patients provided written consent for the use of anonymized data for treatment evaluation and research. For publication of the case description, the patient’s specific consent was obtained. Of the 16 patients who started the treatment programme, three stopped during the first phase of the treatment. Of these, one participant stopped the treatment before standardized measurements were taken. In this study 15 participants completed at least one TGI-SR assessment. Of these, 14 completed at least one CAPS assessment, 13 completed the full treatment as well as both TGI-SR assessments, and 12 completed both CAPS assessments. The initial CAPS interview was delayed until phase 3 in one patient because she felt emotionally overwhelmed during phase 1, therefore no second CAPS interview could be done. Treatment attendance was registered on a weekly basis and therapist impressions were evaluated in a focus group with therapists who offered the BEP-TG and other forms of therapies during the treatment programme. This focus group was led by a colleague experienced in leading focus groups, who was not a member of the treatment team. The topics discussed were practicability and perceived effectiveness. To illustrate the treatment programme and clinical feasibility, a clinical case vignette will be described.

### 1.3. Instruments

#### 1.3.1. Clinician-Administered PTSD Scale

The Clinician-Administered PTSD Scale for DSM-IV (Blake et al., 1995) is a clinician rated interview that taps all diagnostic criteria for PTSD of the Diagnostic and Statistical Manual of Mental Disorders, fourth edition (DSM-IV) (American Psychiatric Association [A.P.A.], 2000). It has excellent psychometric properties across a wide variety of clinical research settings and trauma populations and is the standard criterion measure in the field of psychotrauma for its convergent and discriminant validity, diagnostic utility and sensitivity to clinical change (Weathers, Keane, & Davidson, 2001). A decrease in 15 or more points on the CAPS is considered a clinically significant improvement in PTSD severity whereas CAPS total scores exceeding 80 indicate extreme PTSD (Weathers et al., 2001). Thus, consistent with prior research (Weathers et al., 2001), based on their CAPS scores, patients were categorized into ‘extreme PTSD’ (a DSM-IV diagnosis of PTSD and a CAPS total score ≥ 80), ‘PTSD’ (a DSM-IV diagnosis of PTSD and a CAPS total score < 80), and ‘no PTSD’.

#### 1.3.2. Traumatic Grief Inventory

The Traumatic Grief Inventory – Self Report (TGI-SR; Boelen & Smid, 2017) is a newly developed self-report instrument, measuring lifetime losses of loved persons and assessing current symptoms of PCBD according to DSM-5 (American Psychiatric Association [A.P.A.], 2013). With patients who had experienced multiple losses, the loss that they considered the most painful was taken as the anchor event. It contains 18 items to assess the intensity of grief reactions that are answered on a 5-point Likert scale, ranging from ‘1 = never’ to ‘5 = always’. A diagnosis of PCBD according to DSM-5 was assigned to patients who scored 4 (‘often’) or 5 (‘always’) on (1) at least one core symptom, (2) at least 6 out of 12 additional symptoms, and (3) the dysfunction criterion. A preliminary cross-sectional validation of the self-report version of the Traumatic Grief Inventory (Boelen & Smid, 2017) showed high internal reliability (Cronbach’s alpha = 0.95) and adequate psychometric properties for a screening diagnosis of PCBD.

### 1.4. Statistical analysis

Statistical analyses were performed using SPSS Statistics version 20 (IBM, Armonk, N.Y., U.S.A.). Changes in PTSD symptoms during treatment were calculated for all participants who completed at least one assessment (intent to treat) as well as for those who completed the treatment. For the intent-to-treat analyses, the last observation was carried forward. We used paired t-tests for testing changes in continuous measures (i.e. PTSD severity) and calculated the effect size using Cohen’s d. Diagnostic status with regard to traumatic grief was operationalized as a categorical outcome variable that could take on the following categories: PCBD and extreme PTSD; extreme PTSD only; PCBD and PTSD; PTSD only; PCBD only; or no PTSD or PCBD. To assess whether a statistically significant change in diagnostic outcome proportions had occurred at the two time points, we applied the marginal homogeneity test (Agresti, 2003). The significance threshold was set at p < .05.

## 2. Results

### 2.1. Participant characteristics

In Table 1 participant characteristics are presented. Each participant had experienced multiple traumatic as well as non-traumatic losses and multiple different types of traumatic events. Additionally, about half of the participants reported missing relatives. Countries of origin were Armenia, Bosnia-Herzegovina, Guinea, Hungary, Iraq, Sierra Leone and Syria. Loss and traumatic events characteristics are presented in Table 2. Time since most painful loss ranged from 1 to over 35 years. During the treatment all participants were faced with a variety of ongoing stressors, including receiving negative immigration decisions, illness or death of family members, missing family members or friends and insecurity about the wellbeing of family members left behind in unsafe conflict areas.

**Table 1.** Demographic and clinical characteristics at start of treatment.

		M	(SD)	N	(%)
Age		39.7	(8.8)		
Gender					
	Female			5	(31.3)
	Male			11	(68.8)
Marital status					
	Married			5	(31.3)
	Divorced			3	(18.8)
	Single			8	(50.0)
Residential status					
	Residence permit			2	(12.5)
	Dutch passport			9	(56.3)
	Asylum seeker			4	(25.0)
	Undocumented			1	(6.3)
Employment					
	Employed			1	(6.3)
	Sick leave			4	(25.0)
	Disabled			4	(25.0)
	Unemployed			7	(43.8)
Psychiatric comorbidity^a^					
	Depressive disorder			13	(81.3)
	General anxiety disorder			1	(6.3)
	Dissociative disorder			2	(12.5)
	Substance abuse			1	(6.3)

Note: ^a^Total N adds up to more than 16 due to multiple comorbidity.

**Table 2.** Loss and traumatic event characteristics at start of treatment.

		M	(SD)	N	(%)
Number of traumatic events		9.5	(2.4)		
Number of losses		5.1	(2.5)		
Number of violent losses		2.4	(1.6)		
Participants with missing relatives				8	(53.3)
Relationship to violently lost loved one(s)^a^					
	Partner			2	(13.3)
	Child			3	(20.0)
	Parent			9	(60.0)
	Sibling			9	(60.0)
	Friend			5	(33.3)
	Other			3	(20.0)
Context of loss^a^					
	Murder/War-related			12	(80.0)
	Accident			4	(26.7)
	Suicide			0	(0.0)
	Illness			3	(20.0)

Note: ^a^Total N adds up to more than 15 due to multiple losses per participant, with some of the losses occurring within the same contexts.

### 2.2. Case description

George, now 37 years old, from Liberia, had lived in the Netherlands since 2001 and lived undocumented at the start of the treatment. He was referred for treatment because of PTSD and comorbid depression. At the age of 13 his younger sister was killed. When he was 19 his parents were murdered by the rebels. George was forced to witness their murder. He was captured by the rebels and then forced to be a child soldier. George did not know what happened to the bodies of his parents. George had recurrent nightmares and flashbacks in which he relived the murder of his parents. When he tried to fall asleep at night, intrusive memories of the murder of his parents kept him awake for hours. The nightmares caused feelings of unsafety and confusion as well as anxiety and panic attacks. He had difficulties accepting the death of his parents and had strong feelings of guilt. He found his life meaningless without them. In the days surrounding the anniversary of his parents’ death, he experienced acute physical pain. George mistrusted other people and experienced a lot of anger as well as unresolved feelings of revenge. In moments of stress he was afraid of losing control, which lead to social isolation. George experienced intense distress in response to reminders of traumatic events. He tried to avoid thoughts and memories of being a child soldier, and the way his parents died. George reported a depressed mood, loss of interest in former activities, and no sense of a future. George was diagnosed clinically with PCBD, PTSD and severe MDD. On the CAPS his score was 103, indicating extreme PTSD. On the TGI-SR, his score indicated PCBD.

At the start of treatment George did not feel comfortable about bringing a friend with him to the first therapy session. Information about the treatment was provided and expression of emotions was encouraged. George was afraid of losing control and experienced a lot of anxiety about expressing his grief. Explaining the influence of avoidance on symptom maintenance helped him to engage in the treatment. George talked about his safe and happy childhood and his warm relationship with his parents. With gradual exposure he talked in detail about the day his parents were murdered and how he was taken by the rebels afterwards. In between sessions he had a hard time and got support from a friend as well as his religion. George wanted to write a letter to the rebels about his anger and feelings of revenge, but he felt too overwhelmed. He decided to write letters to his parents about how he was doing and how he felt about them. First this was very difficult for him but in the end he told that the writing of these letters had given him inner peace. Because he was doing better in that period of treatment the therapist decided to support the decision to not write the letter to the rebels. George expressed more of his feelings and told about his struggle to find meaning in their death. He contemplated what his parents would have said about his current life and what advice they would have given him. This contributed to integrating the memory of his parents in a helpful manner.

During the treatment, George obtained a residence permit. As this gave him the opportunity to go back to Liberia, he started planning to visit the places he had lived with his parents and the place where they had died. He wanted to talk to people in that area and see if he could find out what had happened to his parents’ bodies. He wanted to give them a proper burial. As a closing ritual of the therapy he made plans to go to a church there and leave the letters to his parents there behind. Because of safety concerns he had to postpone his plans.

George was satisfied with the treatment. He was able to talk about his parents and felt less preoccupied with their death. Thinking of their death could still make him angry but his anger did not overwhelm him. Instead of avoiding thoughts about his parents, he was now able to integrate a sense of what they would think and say to him in his daily life, which he experienced as very helpful. His CAPS score dropped from 103 pre-treatment to 56 post-treatment, a clinically significant improvement (Weathers et al., 2001). His TGI-SR scores indicated that a diagnosis of PCBD was no longer applicable (Boelen & Smid, 2017).

### 2.3. Changes in PTSD symptoms during treatment

Table 3 shows the changes in PTSD symptom severity (summed CAPS score) during treatment. After treatment, there was a significant decrease in CAPS total scores with a large effect size (d = 0.93 and d = 1.10 according to intent-to-treat and completer analyses, respectively), and N = 8 (66%) reported a clinically significant decrease (≥ 15 points). On a symptom cluster level, avoidance/numbing decreased significantly with a large effect size, and re-experiencing and hyperarousal symptoms decreased on a trend level (p < .10) with a moderate effect size.

**Table 3.** Change in PTSD severity during treatment.

	Time 1		Time 2		Change						
	M	SD	M	SD	M		95% CI		t	p	d
Intent-to-treat sample											
Reexperiencing	31.79	(4.21)	28.36	(6.02)	3.43	(−0.53	–	7.39)	1.87	0.084	0.66
Avoidance	36.86	(6.47)	28.57	(11.30)	8.29	(2.00	–	14.57)	2.85	0.014	0.87
Hyperarousal	30.14	(5.90)	26.64	(5.61)	3.50	(−0.13	–	7.13)	2.08	0.057	0.61
CAPS-I V PTSD^a^ Total Score	98.79	(10.76)	83.57	(18.47)	15.21	(6.32	–	24.10)	3.70	0.003	0.93
Completers sample											
Reexperiencing	31.17	(4.11)	27.17	(5.56)	4.00	(−0.63	–	8.63)	1.90	0.084	0.82
Avoidance	37.33	(6.79)	27.67	(11.96)	9.67	(2.55	–	16.78)	2.99	0.014	0.95
Hyperarousal	30.83	(4.76)	26.75	(4.73)	4.08	(−0.14	–	8.30)	2.13	0.057	0.86
CAPS-IV PTSD^a^ Total Score	99.33	(9.81)	81.58	(18.29)	17.75	(8.09	–	27.41)	4.05	0.002	1.10

Notes: ^a^CAPS-IV: Clinician-Administered PTSD Scale for DSM-IV; PTSD: Posttraumatic Stress Disorder.

### 2.4. Changes in diagnostic status during treatment

Table 4 shows the diagnostic results at the two assessment times. Of the 11 participants with initial PCBD and extreme PTSD, n = 2 (18%) showed full remission of PCBD and PTSD, n = 2 (18%) showed remission of PCBD as well as a decrease in PTSD severity, n = 2 (18%) showed a decrease in PTSD severity, and n = 5 (46%) maintained their diagnostic status. Of the three participants with initial extreme PTSD without PCBD, n = 1 (33%) went on with same diagnosis, n = 1 (33%) could not be assessed a second time, and n = 1 (33%) dropped out of treatment. The changes in diagnostic status were evaluated using marginal homogeneity (MH) tests. For the intent-to-treat sample (N = 14) and the completers sample (N = 12), MH test results were identical: standardized MH statistic = 2.29, p = .022, indicating a significant change in diagnostic status across the two assessment times.

**Table 4.** Change in diagnostic status during treatment.

	Time 1		Time 2	
Diagnosis	N	(%)	N	(%)
PCBD and extreme PTSD ^a^	11	(91.7)	5	(41.7)
Extreme PTSD only	1	(8.3)	1	(8.3)
PCBD and PTSD	0	(0.0)	2	(16.7)
PTSD only	0	(0.0)	2	(16.7)
PCBD only	0	(0.0)	0	(0.0)
No PTSD or PCBD	0	(0.0)	2	(16.7)

Notes: N = 12 patients who completed the treatment; ^a^PCBD: Persistent Complex Bereavement Disorder; PTSD: Posttraumatic Stress Disorder.

### 2.5. Treatment completion

Each day of the treatment programme was attended by a mean of 76% of the participants. Treatment attendance during the individual BEP-TG treatment in phase 2 ranged from 59 to 96%. Treatment completion was 81%: of the 16 patients who started the treatment programme, three stopped during the first phase of the treatment. Of these, two dropped out due to resuming work and one for other reasons.

### 2.6. Results of the focus group

The focus group consisted of 12 therapists and was held in February 2015 after the first two groups of participants completed the treatment programme. Topics discussed were the practicability and perceived effectiveness of the treatment programme. Therapists noted symptom reduction and improvement of daily functioning and regarded BEP-TG as an effective therapy for their patients. According to the focus group members, *‘Symptom reduction can be clinically observed, patients become more active and report experiencing the world as more real.’* Although the therapists recognized acute social stressors and concerns about family members in countries of origin where wars were ongoing as being capable of exacerbating symptom severity, they still considered BEP-TG feasible because they felt that the day patient treatment programme as a whole was perceived as supportive.

## 3. Discussion

The Day Patient Treatment for Traumatic Grief is an innovative treatment for patients with complex problems who, following one or several traumatic losses and other traumatic experiences, are diagnosed with extreme PTSD and/or PCBD and comorbid psychopathology. They have severe impairments in daily functioning and are at risk of being exposed to new and ongoing stressors. The treatment comprises a weekly five-hour programme consisting of three phases with a duration of four months each. Of the first 16 patients referred for the treatment, 13 (81%) completed it, and the mean treatment attendance was 76%. In the first phase of treatment, patients scored very high on PTSD symptoms (mean CAPS score of 98.79). Patients showed significant declines in PTSD symptoms as well as in diagnosable PTSD and PCBD from the first to the last phase of treatment. In the focus group, therapists noted symptom reduction and regarded BEP-TG as an effective therapy for their patients. These preliminary data support the feasibility and potential effectiveness of the day patient treatment programme in a refugee population faced with multiple traumatic losses.

Despite significant symptom reduction with moderate to large effect sizes, several participants still reported high levels of symptoms during the last treatment phase. In a systematic review of treatment efficacy in refugees with PTSD (Nickerson et al., 2011) only few small randomized controlled trials were found. The only RCT using CAPS for measuring symptom severity reported less severe symptoms both pre- and post CBT-treatment, with mean scores of 74.85 and 39.25, respectively (Hinton et al., 2005). Notably, in a meta-analysis of PTSD treatment efficacy in the military (Haagen, Smid, Knipscheer, & Kleber, 2015), high symptom severity pre-treatment was predictive of smaller treatment effects compared with moderate symptom severity pre-treatment.

There are several reasons why refugees benefit less from psychotherapy compared to nonrefugee patients. First, refugees are more likely to suffer from *ongoing stressors* that – combined with their complex psychiatric symptoms – can have a detrimental effect on their daily functioning and that can challenge treatment outcome (Craig, Sossou, Schnak, & Essex, 2008; Nickerson et al., 2014; Steel et al., 2011). Legal procedures, being separated from family and socioeconomic disadvantages, may all contribute to increased symptom severity and block symptom reduction (Schock, Böttche, Rosner, Wenk-Ansohn, & Knaevelsrud, 2016; Schock, Rosner, & Knaevelsrud, 2015; Steel et al., 2009, 2011). Additionally, ongoing stressors can cause distraction and interfere with focus of treatment and motivation for treatment. Research in non-clinical populations has shown that extreme exposure to traumatic events may increase sensitivity to post-trauma stressors, a process that has been termed stress sensitization (Smid, Kleber, Rademaker, Van Zuiden, & Vermetten, 2013; Smid et al., 2012). Given the high reported numbers of traumatic events in our sample, patients may likely experience high levels of distress when confronted with everyday life stressors. Adaptation to new life events and stressors seems to challenge refugees. Nickerson et al. (2014) found that after exposure to trauma and loss, different post-migration living difficulties are associated with specific symptom patterns regarding PCBD and PTSD.

Second, the influence of *multiple losses* on symptom severity has hardly been investigated, but it seems reasonable to assume that a higher number of losses is associated with increased severity of PCBD symptoms. Indeed, one study (Stammel et al., 2013) found a significant association between number of close family members lost and symptom levels of PCBD, although other studies did not find such associations (Morina, Rudari, Bleichhardt, & Prigerson, 2010; Schaal, Jacob, Dusingizemungu, & Elbert, 2010). Also the *relationship to the deceased* may be associated with severity or persisting of symptoms of traumatic grief, although until now few studies among refugees have taken relationship with the deceased into account (Stammel et al., 2013). Future studies are needed to gather more knowledge about this.

Third, *ambiguous loss* may complicate grieving and challenge therapists. Focus of treatment often needs shifting towards dealing with feelings of ambiguity (Boss, 2006). Ambiguous loss may be associated with severe mental health distress comparable to confirmed traumatic losses (Lenferink, de Keijser, Wessel, de Vries, & Boelen, 2017), and symptoms following ambiguous loss have been found to be more severe in relatives holding on to hope that the loved one will return (Heeke, Stammel, & Knaevelsrud, 2015). Future research should take ambiguous loss into account as a moderating factor on symptom severity in patients with traumatic grief.

### 3.1. Study strengths and limitations

The current study examined the feasibility of a treatment programme for this target group with complex mental health problems. We used multiple feasibility indicators, including a case vignette, outcome indicators related to both PTSD and PCBD and process indicators (treatment attendance and therapist satisfaction). Although our results show preliminary significant and positive treatment response, the efficacy of the day patient treatment for traumatic grief in reducing symptoms of PTSD, PCBD and comorbid disorders following traumatic loss (e.g. major depression) needs to be further explored. Also, it should be noted that uncontrolled trials are possibly at risk of overestimating treatment effect.

Our day patient treatment programme started as a treatment programme for a severely distressed patient group, benefitting insufficiently from outpatient care. Different therapeutic interventions were applied during the day patient treatment. The treatment did not start as a research programme, thus we used the measurements which were already part of our general patient care. With the current study design, it is not possible to evaluate the effect of different aspects of the day patient treatment programme separately. Therefore, the data should be seen as preliminary and explorative. Future research is needed to disentangle the effects of the different treatment interventions.

### 3.2. Implications for research and practice

At present, research regarding multimodal treatment of refugees is scarce. In different studies regarding multimodal treatments, no significant improvements were found (Nickerson et al., 2011), possibly due to illness severity and chronicity within this group. Further research of evidence from informed, tailored multimodal interventions is therefore important to elucidate their effectiveness and efficacy. Future research may also elucidate possible predictors of symptom severity and treatment results. Finally, future research may compare the effects of multimodal day patient treatment with those of individual outpatient treatment.

In refugees with PTSD, comorbid PCBD often goes unrecognized. Indeed, many of the patients had gone through previous treatment with limited results and were regarded as patients with complex, chronic, treatment resistant PTSD. Over the course of the day patient treatment programme, symptom reduction and improved daily functioning occurred in most patients. The multimodal group format addressed social stressors and offered support. In addition, treatment attendance was high and patients seemed satisfied with treatment. This study shows that a day treatment programme for refugees with a focus on trauma and loss is feasible and offers preliminary support for its potential effectiveness.

## Highlights

- Refugees referred for specialized treatment because of extreme PTSD often report multiple traumatic losses as well as a variety of traumatic events.
- A group-based multidisciplinary day patient treatment programme embedding individual traumatic grief-focused therapy was developed for refugees.
- Supporting the feasibility of the treatment, significant decreases in PTSD severity as well as diagnosable PTSD and PCBD were observed.
